# Progress in Tropical Diseases

**Published:** 1902-12-20

**Authors:** 


*(To be continued.)*

### Progress in Tropical Diseases.

**Beriberi.**—In opening a discussion on this subject,[1] Manson points out that owing to our ignorance of the true cause of the disease diagnosis must frequently be confused. The chief feature, multiple peripheral neuritis, is a symptom rather than a disease, and beriberi, although distinct from, may occur alongside of cases of peripheral neuritis due to other causes, such as alcohol, arsenic, or malaria. With regard to arsenic being found in the hair of some beriberics, it is pointed out that this drug is frequently used in the arts and in preparing tobacco in some countries where the disease prevails, and is not uncommonly exhibited medicinally in the later stages of the disease. Broadly, Manson distinguishes beriberi as a multiple peripheral neuritis of unknown origin, occurring endemically and epidemically, showing a proneness to produce cardiac disability and dropsy, and frequently a high rate of mortality, but not tending to implicate the skin, the higher nerve centres, nor the cranial nerves, with the exception of the pnenmogastric. Acknowledging that it is, as yet, unproved that only one form of neuritis is included under the head of beriberi, Manson holds that this neuritis is produced by a toxin, the product of a living germ operating in some culture medium outside the human body, and that this toxin is not introduced into the body by means of food or water, but enters through the skin or by inhalation. The fact that beriberi cases recover if moved sufficiently early from the endemic area, points to the infection being due to a toxin, which is gradually eliminated when the dose is stopped, rather than to a germ living within the body of the patient, which would be transferred with him. The bacterium regarded by Pekelharing and Hinkler as the specific cause is now discredited, and recent very careful work by A. Stanley, of Shanghai, has failed to find any organism in the blood of beriberi patients. That the toxin is elaborated by a living germ is regarded as proved by the fact that the disease has been introduced and spread in virgin countries. A recent notable instance is quoted, reported by J. Bolton, of Mauritius, as occurring in Diego Garcia, a small island adjacent to Mauritius. An analogy is drawn to the production of a poison alcohol, elaborated by a germ—yeast, in a culture medium—a saccharine solution, outside the body, but in the case of beriberi poison, germs, and culture medium are alike unknown. Manson regards it as proved that neither water nor food convey the poison to man. In proof of the latter assertion detailed reference is made to a recently published paper by Travers, of Kuala Lumpor, Malay, recording some observations made by him in 1895. In the old gaol at Kuala Lumpor, beriberi had been unknown for many years, but on the transference of the prisoners to a new gaol, the Pudoh Gaol, beriberi of a fatal type speedily broke out and has stuck to the place ever since. On transferring the cases as they appeared to the old gaol, the mortality rapidly fell. Both gaols and three other public institutions were being supplied with food, or at any rate, with rice of the same kind and equality, and all the rice was obtained from the same contractor, but only in the Pudoh Gaol, situated a mile and a half from the old gaol, did any case of beriberi arise. By way of experiment for two and a half months the whole of the food used at the old gaol was supplied by and cooked in the Pudoh Gaol, and subsequently conveyed to the old gaol for consumption by the residents there. Not a single case developed in the old gaol notwithstanding. Manson regards this as very strong evidence against the theory of food or rice conveyance of the disease. E. R. Rost adheres strongly to the rice theory of causation, and states that he has found in rice-water liquor and in mouldy rice an "angular sporulating diplobacillus," which was also present in the blood and cerebro-spinal fluid of a large number of beriberi cases. He produced an exactly similar disease in fowls—a disease characterised by anæmia, loss of feathers and weight, diarrhœa, weakness, paralysis, cyanosis, and death—by: (1) Feeding them on fermenting rice obtained from rice-liquor shops; (2) feeding them on mouldy rice obtained from the lower bags of damp godowns; (3) intraperitoneal injection of rice-water liquor; (4) subcutaneous or intraperitoneal injection of the venous blood of beriberi patients; (5) reinjection from fowls suffering from the disease produced as above. Post-mortem hyperæmia and thickening of the gastrointestinal tract were found, and in some cases petechiæ in the small intestine. He concludes from his experiments that beriberi must be caused by a micro-organism in the blood, which is the same as that produced by eating bad rice, since the symptoms produced by the ingestion of bad rice are identical in fowls with those produced by the injection of the blood of beriberi patients. L. W. Sambon also thinks that the weight of evidence in favour of the rice theory cannot be overthrown. He thinks that some micro-organism which causes the disease may sometimes be associated with rice, and perhaps with other grain, and inclines to the belief that all forms of multiple peripheral neuritis are due to a specific organism, and that the administration of alcohol or arsenic may act by favouring the development of this organism, just as the infection of typhoid fever, influenza, diphtheria, syphilis or other disease may favour their development. He believes "that the specific agent of beriberi lives within the patient's body and attacks the peripheral nerves." J. Cantlie points out that beriberi does not arise from scanty food. In the well-known case of the Japanese navy improved hygiene was introduced coincidently with improved food, and in certain barracks where the hygiene was improved, but the food remained unchanged, the disease disappeared as quickly as in those where improvement in both was carried out simultaneously. In the Dutch Indies it was found that improved food was ineffectual in staying the disease until supplemented by improved hygiene. In a ward in Hong Kong, containing 16 beds filled with medical and surgical cases, to which three beriberi cases were admitted, three other cases developed the disease. All three were suffering from chronic ulcer of the leg, and each happened to have a medical case on either side. W. T. Prout relates how 250 negroes were landed at Sierra Leone, suffering from beriberi. Rice is the staple article of food here, but beriberi is not endemic. These 250 patients were placed under canvas in the open air and fed well. Not a single case occurred in the town, and no case developed among the negroes after landing. R. Ross publishes another series of cases in which arsenic was found in the hair of beriberi patients. It appears to be found in the early days of the disease. Out of 29 cases examined, arsenic was present in nine, and of these eight were cases of under one month's duration. Of the remaining 20 cases, in 18 the disease had lasted more than one month.

1 Brit. Med. Jour., Sept. 20.

